# Biomaterials and Cell-Based Regenerative Therapies for Intervertebral Disc Degeneration with a Focus on Biological and Biomechanical Functional Repair: Targeting Treatments for Disc Herniation

**DOI:** 10.3390/cells11040602

**Published:** 2022-02-09

**Authors:** Katsuhisa Yamada, Norimasa Iwasaki, Hideki Sudo

**Affiliations:** 1 Department of Orthopaedic Surgery, Faculty of Medicine and Graduate School of Medicine, Hokkaido University, Sapporo 060-8638, Japan; yka2q@yahoo.co.jp (K.Y.); niwasaki@med.hokudai.ac.jp (N.I.); 2 Department of Advanced Medicine for Spine and Spinal Cord Disorders, Faculty of Medicine and Graduate School of Medicine, Hokkaido University, Sapporo 060-8638, Japan

**Keywords:** intervertebral disc degeneration, regenerative therapy, biomaterial, cell transplantation, disc herniation

## Abstract

Intervertebral disc (IVD) degeneration is a common cause of low back pain and most spinal disorders. As IVD degeneration is a major obstacle to the healthy life of so many individuals, it is a major issue that needs to be overcome. Currently, there is no clinical treatment for the regeneration of degenerated IVDs. However, recent advances in regenerative medicine and tissue engineering suggest the potential of cell-based and/or biomaterial-based IVD regeneration therapies. These treatments may be indicated for patients with IVDs in the intermediate degenerative stage, a point where the number of viable cells decreases, and the structural integrity of the disc begins to collapse. However, there are many biological, biomechanical, and clinical challenges that must be overcome before the clinical application of these IVD regeneration therapies can be realized. This review summarizes the basic research and clinical trials literature on cell-based and biomaterial-based IVD regenerative therapies and outlines the important role of these strategies in regenerative treatment for IVD degenerative diseases, especially disc herniation.

## 1. Introduction

Intervertebral disc (IVD) degeneration is a common cause of low back pain that affects the daily life of afflicted individuals and is the cause of most spinal disorders [1,2,3,4]. Surgical treatments, such as discectomy, arthroplasty, and spinal fusion, have been widely used for the treatment of IVD diseases, including disc herniation, spinal canal stenosis, and spinal deformities. However, these methods limit spine mobility and fail to maintain the function of the treated IVD for extended periods of time [5]. Furthermore, the appearance of postoperative adjacent intervertebral disorders and reports of functional impairment are widely recognized. Meanwhile, conservative treatments for low back pain, such as the administration of analgesics, are only symptomatic therapies and do not focus on the underlying etiology [4]. In other words, there is currently no clinical treatment that can prevent or reverse IVD degeneration. Fortunately, recent tissue engineering approaches have revealed the molecular cascade involved in IVD degeneration, and treatments aimed at the regeneration of degenerated or damaged IVDs have been attempted [5]. Specifically, cell-based therapies and biomaterial-based regenerative therapies have recently attracted attention as therapeutic methods to prevent or repair IVD degeneration.

IVDs consist of an inner gel-like structure, nucleus pulposus (NP), and external annulus fibrosus (AF) [6]. The extracellular matrix (ECM) of gelatinous NP is composed of glycosaminoglycans, proteoglycans (PG), and type II collagen, which are highly hydrated [7,8]. The function of the NP is to distribute the hydraulic pressure under compressive loads [7,8]. IVD degeneration is characterized by a loss of hydration and degradation of the ECM of the NP with NP degeneration leading to overall changes in the biomechanics of the spine [5,7,8,9]. Therefore, regenerative therapy of the NP is a promising strategy to restore the function of IVDs that exhibit less advanced deformities [4,7,10,11]. 

**Figure 1:**
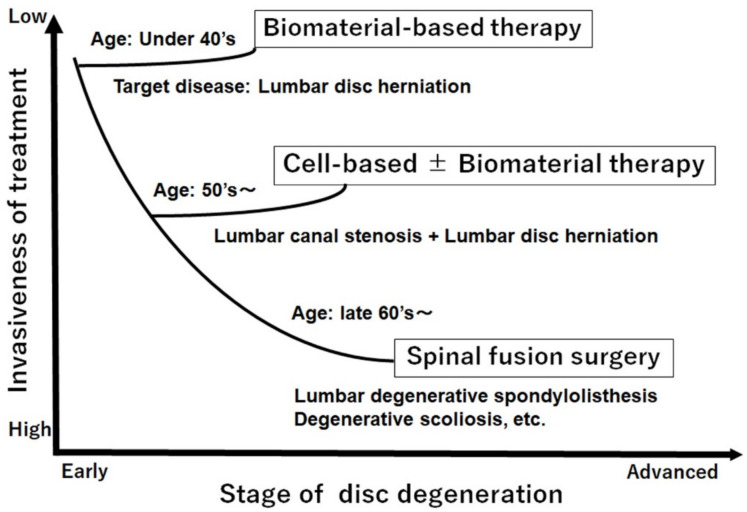
Biomaterial-based and cell-based intervertebral disc regeneration treatment strategies based on the stage of disc degeneration.

Since the degree of IVD degeneration generally reflects the regenerative capacity of the disc, treatment strategies for IVD regeneration are also based on the severity and stage of IVD degeneration [12]. In the early stages of disc degeneration, the structural integrity of the disc is preserved, and there are many surviving native disc cells; therefore, biomolecular and genetic engineering interventions may have a regenerative effect [12]. In the intermediate stages of degeneration, the number of surviving native disc cells decreases, and the structural integrity of the disc begins to disintegrate [12]. Among the potential interventions, biomaterial-based therapies that maintain the disc structure and activate the remaining cells are expected to be applicable to degenerative disc diseases, such as lumbar disc herniation, which typically affects relatively young people up to the age of 40 years (Figure 1). In degenerative disc diseases, such as lumbar disc herniation combined with lumbar spinal canal stenosis, which tends to occur in people over 50 years of age, the number of remaining cells is further reduced. Therefore, cell-based therapies that repopulate the disc with healthy cells that may restore normal tissue homeostasis, in addition to the combination of biomaterials with cell therapies, are expected to be effective in these situations (Figure 1). Multiple clinical trials have been conducted to investigate cell-based and biomaterial-based therapies to treat IVD degeneration, with each trial being supported by numerous preclinical animal studies and basic science experiments [12].

In this article, we review the strategies for disc regeneration/repair therapy for IVD degeneration, with a focus on cell-based therapy and soft biomaterial-based approaches. This includes

1. cell-based IVD regeneration therapy,
2. biomaterial-based IVD regeneration therapy, and
3. disc regeneration/repair treatment for IVD herniation.

To prepare this review, 231 English-language articles were extracted from PubMed searches using the keywords “intervertebral disc degeneration” AND “regenerative therapy” without a date limitation (start of the database through October 2021). Additional keywords (biomaterials, cell transplantation, disc herniation, biomechanical, biological) were used to narrow down the papers to the topics most relevant to this review. Moreover, additional papers were obtained by analyzing papers containing excellent reviews.

## 2. Cell-Based IVD Regeneration Therapy: Cell Transplantation

Apoptosis of the NP cells is a characteristic phenomenon that occurs in the early stages of IVD degeneration [4,11,13,14]. NP cells play an important role in the synthesis of ECM proteins that maintain IVDs. Aging and degeneration of the IVD result in a decrease in the number of NP cells and a reduction in the production of ECM [4,13,15,16,17,18]. As the number of viable NP cells is reduced in the early to intermediate stages of disc degeneration, intradiscal cell therapy (e.g., stem cells or chondrocytes) can be used to restore normal tissue homeostasis to the disc and repopulate the disc with healthy cells (Figure 1).

There is increasing evidence that supports the use of biological and cell-based therapies for IVD degeneration. Several cell sources, including IVD-derived cells (NP-derived cells), chondrocyte-like cells, mesenchymal stem cells (MSCs), induced pluripotent stem cells, and embryonic stem cells, have been proposed and evaluated for disc regeneration therapy via cell transplantation [12,19,20] (Table 1). Several clinical trials have also been conducted using these cells for IVD degeneration [12,19,21,22,23,24] (Table 2).

### 2.1. Autologous IVD-Derived Cells as Therapy for IVD Regeneration

Transplantation of autologous NP-derived cells is physiologically more natural than allogenic transplantation, and autologous transplantation can avoid graft-versus-host reactions [25]. NP cells have the ability to survive in the harsh, bloodless environment of the IVD and are able to produce IVD-specific ECM [12]. However, harvesting autologous cells from a patient’s disc is invasive, and the cell yield is relatively low because the tissue source is compromised [19]. Furthermore, the ability of NP cells isolated from degenerated NPs alone is insufficient to delay further disc degeneration [25]. The co-culture of NP cells with MSCs, which allows for direct cell-to-cell contact, has been shown to significantly improve the viability of NP cells [26,27]. In a pilot clinical trial, these co-cultured cells were transplanted into a degenerated lumbar disc at a level adjacent to the fusion surgery segment. Three years post-surgery, there was no progression of disc degeneration and no back pain, suggesting the minimal efficacy needed to slow the further degeneration of human IVDs [25]. In the EuroDISC study in which percutaneous transplantation of autologous disc cells was investigated, disc chondrocytes harvested at the time of discectomy were grown in vitro and then injected into patient discs at three months postoperatively [28]. In the 2-year analysis, patients had significantly less back pain compared with that of the control patients, and NP fluid levels on magnetic resonance imaging (MRI) remained higher at the treated and adjacent discs [28].

### 2.2. MSC Therapy for IVD Regeneration

MSCs are the most common clinically evaluated cell type for disc regeneration therapy. MSCs are undifferentiated somatic cells that are capable of self-renewal and have the potential to differentiate into any lineage of mesenchymal origin, including chondrogenic and IVD-cell lineages, owing to their ability to differentiate into a variety of mesodermal lineages [12,81,82]. MSCs are thought to resemble perivascular and pericyte cells and are found almost everywhere in the body where vascularity is abundant [81,83]. Accordingly, autologous MSCs can be easily harvested from the bone marrow or adipose tissue [12]. Numerous studies have demonstrated that MSCs promote tissue repair and reduce inflammatory damage, while multiple preclinical animal models have been used to demonstrate the ability of MSCs to differentiate into NP cells, restore disc height and hydration, and inhibit the inflammatory cascade, leading to disc regeneration [12,26,50,67,84,85,86]. Various sources of MSCs have been identified and studied, including the bone marrow, synovial membrane, and adipose tissues [87] (Table 1). A systematic review of the safety and efficacy of MSCs for treating IVD degeneration has shown that three types of MSCs can successfully inhibit IVD degeneration, bone marrow-derived, synovial-derived, and adipose tissue-derived MSCs [87].

Both autologous and allogenic MSCs are being investigated for their ability to regenerate discs by transplanting them into degenerated discs [81]. The use of autologous MSCs as an injectable therapy has been investigated in several completed or ongoing clinical trials [12,81]. Two preliminary clinical studies have reported that transplantation of autologous bone marrow stem cells into the human lumbar disc can improve pain and other clinical outcomes, raise the level of disc stability, and increase disc hydration on MRI [51,52]. In several subsequent clinical trials, patients treated with autologous cultured bone marrow-derived MSCs for IVD degeneration showed significant improvements in pain, function, and overall subjective improvement with minimal adverse events at 4 to 6 years after treatment [58,88]. These studies have demonstrated the long-term efficacy of autologous bone-marrow-derived mesenchymal stromal cell (BM-MSC) therapy in inhibiting disease progression. However, no conclusions have been drawn regarding the optimal conditions of culturing and administrating of MSCs [24]. Although autologous cell transplantation has the lowest risk of immunogenic reactions, it has several limitations for clinical application, including a lack of shelf availability, the need to harvest tissues from patients, the time and expense of cell growth, differentiation, and selection, and the potential risk of infection [19,60,89].

On the other hand, allogeneic MSCs are typically harvested from young, healthy donors and can solve the problem of shelf availability and may produce better long-term functional outcomes as they are not subject to age-related changes and other effects based on the patient’s protoplasm that may occur with autologous cells [22,89]. Animal studies have shown that allogeneic MSCs injected into the NP region of IVDs can survive and proliferate, producing beneficial effects on IVD degeneration [48,57,59,67,89,90,91,92]. In addition, a phase I/II randomized controlled trial examining the therapeutic efficacy of allogeneic BM-MSCs for IVD degeneration confirmed the feasibility and safety of this approach for patients with IVD degeneration [59]. This trial reported rapid improvement in pain and disability for the cell therapy group compared with that for the control group, and improvement in degeneration was observed on MRI. As noted above, MSC transplantation to treat IVD degeneration is able to repair IVD degeneration in patients with low back pain, providing pain relief and functional recovery. However, MSC therapy to treat musculoskeletal disorders has not yet been approved for clinical use by the U.S. Food and Drug Administration (FDA) [78,93]. 

### 2.3. Use of Bone Marrow Aspirate Concentrate (BMAC) for IVD Regeneration Therapy

The use of BMAC has been approved by the FDA to treat musculoskeletal diseases, and its application as an IVD regeneration therapy has been reported [78,79,80]. In an animal study in which a bioresorbable alginate gel containing BMAC was implanted into rabbits of a discectomy model, the BMAC demonstrated regenerative effects on disc degeneration, comparable to that of BM-MSCs [78]. In addition, a prospective clinical trial in which BMAC was injected percutaneously into patients with lumbar disc degeneration showed that disc-derived low back pain can be reduced and disc hydration sporadically increased, demonstrating the usefulness of this treatment [79,80]. Furthermore, BMAC produced using autologous non-cultured cells can be obtained in a single step, which has the potential of cost and time-saving advantages, reduced risk of infection, and a lower chance of sample confusion compared with that of cultured autologous or allogeneic cells [78].

### 2.4. Problems of IVD Regeneration Therapy Using Cell-Only Transplantation

The challenges of IVD regeneration therapy using cell-only transplantation include problems of the transplanted cells adapting to the environment, cell survival, and leakage of the transplanted cells from the injection site. The environment of degenerated IVDs is unsuitable for cell viability and survival, with low oxygen levels, limited nutrition, acidic pH, and high osmolarity, which adversely affect the function and survival of the transplanted cells [65,94,95,96]. Furthermore, the risk of MSCs injected into the IVD leaking from the injection site and causing osteophyte formation has been reported in in vivo studies [45,97]. In a systematic review of MSC-based therapies for IVD degeneration, MSC-related complications were found in 2.7% of all animal models, including osteophyte formation associated with cell leakage [45,87,98].

The usefulness during cell transplantation of combining soft biomaterials as cell carriers and scaffolds, such as alginate, fibrin, atelocollagen, and hyaluronic acid, has been reported [5,26,64,68,92,99,100,101,102]. Hydrogels and other soft biomaterials are expected to not only serve as carriers to hold cells transplanted into IVDs but also act as sealants to prevent cell leakage, thereby improving biomechanical function, protecting the transplanted cells, and even activating the remaining native cells.

## 3. Biomaterial-Based IVD Regeneration Therapy: Soft Biomaterials Used to Regenerate Biological and Biomechanical Function

Although a number of stem cell-based therapies focusing on progenitor cell expansion and transplantation have been investigated as a means of disc regeneration therapy, there are many challenges to their clinical application, including immune rejection, pathogen infection, potential tumor formation, and host tissue engraftment [7,103,104,105,106]. On the other hand, matrix-based medicine using soft biomaterials may provide an alternative single-step process using biomaterials amenable to long-term storage, which can be used for on-demand treatment [7,107]. 

**Figure 2:**
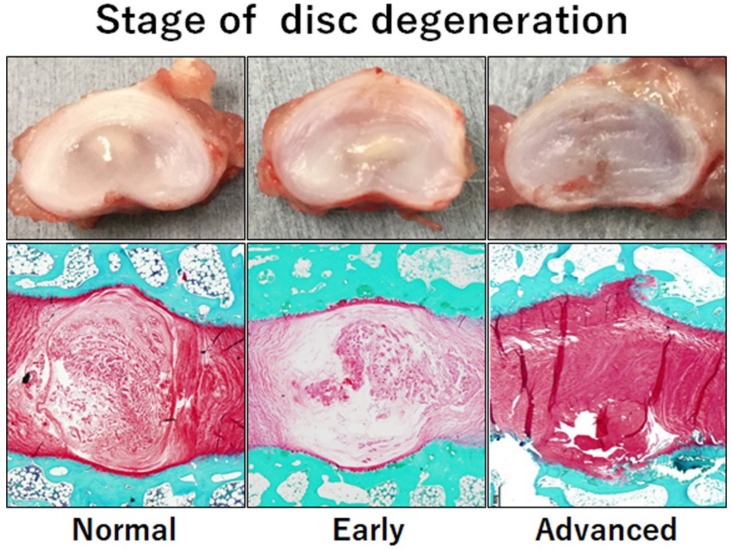
Normal to degenerated lumbar intervertebral discs in rabbits. The initial degeneration of intervertebral discs occurs primarily in the nucleus, making the gelatinous nucleus pulposus a promising target for therapeutic approaches using soft biomaterials.

One goal of disc regeneration therapy is to restore the biomechanical disc function that supports the trunk and maintains mobility. In a healthy IVD, the NP compresses when an axial load is applied to the IVD and transmits the load radially to the AF [81]. Laminated AF has a high tensile strength and can expand radially in response to the added load [81]. There are few cells in the NP (4×10^3^ cells per mm^3^), with NP cells accounting for only approximately 1% of the volume of IVD tissue [81,108,109,110]. The ECM produced by the NP cells is mostly composed of PG, which provides cushioning to the NP by retaining water [81]. As the IVD degradation process progresses, a decrease in PG of the NP reduces the swelling pressure of the disc, resulting in a decrease in the aggregate and instantaneous shear modulus [109,111,112]. In addition, the nucleus loses water and becomes fibrous, with the mechanical properties of the ECM being further impaired [81]. This deterioration leads to reduced flexibility of the NP and alters the loading pattern within the disc, leading to AF delamination [81,109] (Figure 2). Since the initial degeneration of IVD occurs primarily in the nucleus, gelatinous NP appears to be a promising target for therapeutic intervention (Figure 2) [109]. Therefore, tissue engineering using hydrogels and other soft biomaterials may be an alternative to current treatments.

### 3.1. Soft Biomaterials for NP Repair and/or Regeneration

Ideally, biomaterials for NP repair should accommodate both the biological and mechanical aspects of IVD repair and regeneration [109]. The objectives required for a soft biomaterial-based NP repair approach in terms of the biological response include the soft biomaterial

1. being biocompatible, non-toxic, and safe in vivo;
2. support cell survival;
3. promote ECM formation;
4. reduce inflammation; and
5. inhibit pathological fibrosis [113].

In terms of biomechanics, the soft biomaterial should (1) remain within the disc under in vivo loading conditions and (2) improve biomechanical disc function and spinal stability.

The concept of using biomaterials that can be injected into IVDs began in the 1960s when Nachemson et al. [114] proposed a method of injecting vulcanized silicone into degenerated discs for nucleus augmentation. Over the subsequent decades, IVD substitutes have been developed to restore disc function [115]. NP replacement using injectable, in situ curable materials can maintain immediate disc height and mechanical disc weight-bearing capacity [116,117,118,119,120] but is restricted by the risk of complications, such as implant dislocation and endplate damage, and by the limited potential for biological repair [116,117].

### 3.2. Biological NP Repair and/or Regeneration Using Soft Biomaterials

Many polymeric materials have been experimentally investigated for use as NP-regenerative soft biomaterials. Biomaterials are hydrogels or solid scaffolds and can be divided into synthetic biopolymers and natural biomaterials [121] (Table 3). Synthetic materials include poly (D,L-lactide) (PLA) and its derivatives, polyethylene glycol (PEG), polycarbonate urethane (PU), and poly (ε-caprolactone) (PCL), some of which can function as both hydrogels and solid scaffolds [38,63,121,122,123,124,125,126,127,128,129,130,131,132,133,134,135,136,137,138,139,140,141,142,143,144,145,146,147,148,149,150,151,152,153,154,155]. Synthetic hydrogels consist of polymer networks that can absorb a large amount of water, are easy to modify, and can be consistently and highly tunable [121]. However, most production processes of synthetic hydrogels involve the use of reactive reagents and require the complete removal of contaminants and unreacted reagents [115,156]. In comparison, natural polymer-based biomaterials mainly include hydrogels, such as alginate, agarose, fibrin, hyaluronic, collagen, chitosan, and carboxymethylcellulose [4,5,7,39,41,49,57,64,65,66,78,90,92,100,101,116,121,122,128,132,140,142,157,158,159,160,161,162,163,164,165,166,167,168,169,170,171,172,173,174,175,176,177,178,179,180,181,182,183,184,185,186,187,188,189,190,191,192,193,194,195,196,197,198,199,200,201,202,203,204,205,206,207,208,209,210,211,212,213,214,215,216,217,218,219,220,221,222,223,224,225,226,227,228,229,230,231,232,233] (Table 3). These natural hydrogels have been extensively studied for NP tissue engineering due to their excellent biocompatibility and biological activity and their participation in the physiological turnover process [116,122,234]. A number of in vitro studies have shown that these hydrogels support cell survival and induce differentiation of residual NP disc cells and stem cells [132,142,158,159,160,163,169,188,197,203,207,208,232,235,236,237,238].

To achieve intrinsic and sustainable disc regeneration, soft biomaterials are required to support cell survival and induce in vivo differentiation of the transplanted stem cells and remaining disc cells. Hydrogels, such as collagen gel (atelocollagen), hyaluronic acid, fibrin, peptide hydrogel, polysaccharide hydrogel, and alginate, have been reported in in vivo studies to be useful as cell carriers for cell transplantation and disc regeneration therapy [26,57,60,65,68,78,92,196,239]. Degenerated discs present harsh microenvironments characterized by hypoxia, hypotrophy, acidic pH, high mechanical loading, high osmotic pressure, and a complex network of various proteases and cytokines [95,113,240,241,242,243,244]. Meanwhile, biomaterials incorporate cells into the scaffold to increase their viability, act as protective carriers to prevent the leakage of the cells from the site, and also support the transmission of mechanical loading [157]. In addition, several in vivo studies of IVD regeneration with cell-free biomaterials using hydrogels alone have reported the regenerative potential of fibrin sealant, polyglycolic acid (PGA)-hyaluronic acid scaffold, and collagen-based scaffold through hydrolysis with actinidin protease and ultra-purified alginate (UPAL) gel [7,101,107,125,192,204,220].

### 3.3. Mechanism of IVD Regeneration Therapy Using Cell-Free Soft Biomaterials Alone

Considering the various issues related to the clinical application of cell transplantation therapy, biological disc regeneration using cell-free soft biomaterials alone may be a new alternative to the current treatment for disc degeneration disease and ideally involve a single-step process [7,107]. For instance, there has been much interest in bioengineering approaches in recent years that exploit endogenous cell populations to restore the structure and function of IVDs, with the potential for IVD repair using cell-free soft biomaterials being promising [99,220]. Several in vivo studies have shown that various soft biomaterials have the potential to regenerate IVD tissue by supporting the survival and activation of remaining disc cells in damaged or degenerated IVDs and by promoting ECM production. However, details regarding their repair mechanisms have not yet been fully elucidated.

Several biomaterials have been analyzed in in vivo experiments with respect to their mechanisms in inducing and activating residual disc cells (Table 4). For instance, an in vivo rabbit study of IVD aspiration followed by alginate-based hydrogel called UPAL gel implantation revealed a significant increase in the percentage of GD2Tie2 cells [7,92], which are NP progenitor cells [245]. This indicated that the implanted biomaterial was able to induce endogenous NP cells and NP progenitor cells, leading to endogenous IVD repair [7]. Similar to the UPAL gel results, a collagen type 1-based scaffold called low adhesive scaffold collagen (LASCol) promotes internal migration of the remaining disc NP cells when implanted after discectomy of rat caudal IVDs [220]. Furthermore, it has been shown that LASCol promotes the formation of cell aggregative spheroids that facilitate the maintenance of the original disc NP phenotype, upregulates the expression of chondrogenic genes, and promotes disc tissue repair [220].

Biomaterials affecting the expression of various cytokines in damaged discs have also been reported as a mechanism of biomaterial-induced disc repair. For instance, fibrin injection (fibrin sealant) after discectomy of porcine IVD has been shown to suppress acute production of proinflammatory cytokines TNF-α, IL-1β, and IL-6, increase the expression of pro-resolution cytokines IL-4 and TGF-β, and inhibit discectomy-induced progressive fibrosis of NP [192]. Furthermore, hyaluronan treatment after rat tail disc injury regulates inflammation by downregulating IFNα, reduces cell death by suppressing the expression of IGFBP3 and caspase-3 fragment p17, and induces the production of ECM [205].

### 3.4. Effects of Biomaterials on Reduction in Pain Related to Damaged IVDs

The goal of biomaterial-based IVD therapy is to not only inhibit tissue degeneration but also to control the pain caused by disc injury and degeneration. Inflammation within the lumbar IVD is often a key factor in acute low back pain [168,247,248]. Intradiscal inflammation and sensory nerve ingrowth into the deep inner layers of the AF cause discogenic pain during the chronic phase of IVD damage and degeneration [168,249]. Several types of soft biomaterials proposed as candidates for IVD repair have been shown to inhibit inflammatory cytokines in IVDs and are expected to reduce pain. Recently, it was reported in an in vivo rat IVD injury model for which methods evaluating pain-related behavior were established that hydrogels suppress pain [168,209]. Meanwhile, implantation of a hydrogel (hyaluronic acid hydrogel and UPAL gel) in a rat caudal NP punch model inhibited nociceptive behavior in Hargreaves, von Fley, and tail-flick tests [168,209]. The following possible mechanism of the hydrogel effect in the IVD injury-induced pain model has been reported. First, hydrogels implanted into injured discs of rats have been shown to regulate inflammation by inhibiting the downstream signaling cascade that activates nuclear factor κB (NF-κB) and mitogen-activated protein kinase (MAPK) by downregulating IL-6 and IL-β and by inhibiting their binding to receptors [209]. Second, discogenic pain in the chronic phase is caused by an increased expression of nerve growth factor (NGF) that is induced by proinflammatory cytokines and the binding of NGF to its high-affinity receptor, tyrosine kinase A (TrkA), which promotes neoinnervation of the IVD and local inflammation [168,250,251,252]. Third, hydrogel treatment of injured discs has been shown to suppress neurotrophic factors, such as NGF, and reduce NGF-TrkA binding, which mediates inhibition of neurite outgrowth of sensory nerves in the discs, resulting in reduced pain-related behavior in rats [168,209]. Finally, hydrogel treatment of damaged IVDs is expected to have a palliative effect on acute IVD pain after discectomy, as well as a preventive effect on discogenic pain [168].

### 3.5. Biomechanical Evaluation of Soft Biomaterials for NP Repair and/or Regeneration

From a mechanical perspective, soft biomaterials for use in NP treatment should ideally mimic the material properties of NP and withstand physiological loading conditions in order to restore their biomechanical properties [109]. The water content of NP is >85% by weight in juveniles, decreasing to approximately 70–75% in adults, and further decreases with additional aging and degeneration [253,254,255]. The swelling stress and effective, cohesive modulus of non-denatured human NPs in constrained compression tests are 0.138 MPa and 1.01 MPa, respectively [112], and the complex modulus of NP is 5.82 kPa at 1 rad per second, 10% compressive strain in torsional shear tests of the viscoelastic shear properties of NP [109,111]. There have been many in vitro studies on soft biomaterials that mimic the mechanical properties of native NP tissues, including alginate hydrogel, collagen gel, hyaluronic acid hydrogel, and polyethylene glycol hydrogel, among others [115,173,211,256]. It has been shown that these materials exhibit biomechanical properties comparable to those of NPs, such as water content, stiffness, and viscoelastic properties, making them candidate materials for use in NP therapy. These candidate soft biomaterials for NP treatment were first evaluated in vitro and subsequently in situ using ex vivo or in vivo preclinical animal models; however, no consensus has yet been established regarding their biomechanical evaluation as functional spinal units [109,257]. This may change as biomechanical evaluation methods have been proposed to establish best practices for screening the performance of newly developed hydrogel formulations and ensure that these materials meet minimum feasibility benchmarks for translation [257].

In general, biomechanical analysis should include (1) evaluation of the effect of hydrogel on disc function repair for axial, torsional, and viscoelastic motion segment responses and (2) evaluation of durability, mechanical feasibility, and the associated herniation risk [257]. An ex vivo approach using cadaveric animal/human motion segments can be used to investigate the biomechanical suitability of the material(s) under study [109]. Motion segments have been tested under uniaxial compression, lateral bending, and flexion/extension as a biomechanical evaluation of IVD [242]. Meanwhile, the axial compressive properties of IVDs are usually investigated in vertebra-disc-vertebra specimens of the lumbar spine, with the load-displacement curve showing a nonlinear viscoelastic response [242]. In other experiments using uniaxial compression, creep, stress relaxation, vibration/dynamic compression, and high load factor properties have been evaluated [242]. As IVDs are subjected to complex three-dimensional loading in vivo, they should be evaluated on a mechanical spine tester that can apply various combinations of cyclic compression, bending, and torsion to spinal segments ex vivo [242]. Typical moment–rotation graphs reveal marked nonlinearity and hysteresis and can be used to evaluate stiffness, the neutral zone, and range of motion. A setup using six degrees of freedom provides insight into the resulting range of motion and its restoration to previous values [109,242]. Currently, several ex vivo and in vivo studies have reported that soft biomaterials, including alginate, hyaluronic acid, chitosan-based hydrogels, and fibrin, are able to restore biomechanical disc functions, such as stiffness and range of motion, after disc implantation [7,192,215,258].

In contrast, it has been reported that hydrogel injected into IVDs may extrude out of the disc in vivo, with no improvement in biomechanical evaluation [206]. Therefore, to apply hydrogel candidates to preclinical animal models and clinical trials, it is very important to determine in situ IVD repair, a configuration for which there is currently no document to guide the evaluation of the development of new hydrogel systems for IVD treatment, including the evaluation of functional outcomes, such as implant herniation risk and structural durability [257].

Herniation risk following IVD repair has been assessed using a cyclic axial loading test and displacement-controlled ramp-to-failure test [7,230,233,257,259,260,261,262,263]. In particular, the ramp-to-failure test is designed to evaluate the worst-case IVD motion segment failure characteristics and hydrogel sealing properties as the motion segment is compressed with five degrees of side-bending and NP displacement is induced in the radial direction of the hydrogel [257]. Fatigue endurance testing has also been performed using a fatigue loading protocol established by Wilke et al. [264]. In this test, called the hula hoop test, the IVD motion segment is subjected to cyclic eccentric compression at an offset that induces a physiological bending moment until failure is reached, with NP extrusion being examined and flexibility testing being performed [233,257,264]. When assessing the risk of implant herniation and structural durability, the degree of biomechanical recovery should be assessed by comparing the motion segment of the repaired IVD with that of the intact IVD, and the biomechanical non-inferiority or superiority relative to standard treatment should be demonstrated by comparison with an IVD injury model that simulates discectomy [257].

### 3.6. Clinical Trial of Soft Biomaterials for Treating IVD Degeneration

As noted above, numerous in vitro and in vivo experiments on NP regeneration therapy based on using soft biomaterials have been performed, but very few human clinical trials have investigated the use of biomaterials alone or as a cell scaffold or delivery system for IVD regeneration. [24,113]. One of the clinical trials in which fibrin sealant was injected into the IVDs of patients with discogenic low back pain reported on the safety of the treatment with significant improvement in pain and function at a 24-month follow-up [195]. Another preliminary study showed that collagen sponges containing autologous BM-MSCs that were percutaneously transplanted into the discs of two patients resulted in improved hydration and motion segment instability of the degenerated discs and improved low back pain at two years postoperative [51]. Using a fibrin carrier in a clinical trial of 15 patients with lumbar spondylolisthesis associated with mechanical low back pain, allogeneic juvenile chondrocytes were percutaneously injected into degenerated IVDs, resulting in no apparent side effects at 12-month follow-up and significant improvement in disability and pain scores, with 77% of the patients showing improvement on MRI [39]. In a phase I study in which a combination of hyaluronic acid derivatives and autologous adipose tissue MSCs was percutaneously injected into the discs of 10 patients with chronic discogenic low back pain, there were no serious adverse events during a one-year follow-up period, and the patients showed significant improvement in visualized analog scale (VAS) and Oswestry Disability Index (ODI) scores for pain, as well as improved disc hydration on diffusion MRI [64].

## 4. Disc Regeneration and/or Repair Treatment for IVD Herniation

One of the aforementioned targets for NP regeneration therapy is lumbar disc herniation, which is one of the main causes of back pain and is a psychological burden [168,265,266]. A discectomy for a herniated disc relieves pain by removing the NP through fissures in the AF, which relieves nerve compression. However, this procedure does not aim to repair defects in the NP or AF, and the defect within the IVD produced by discectomy can lead to undesirable postoperative outcomes, including further disc degeneration, chronic low back pain, and recurrent herniation [168,257]. To compensate for this defect, disc reparative therapy using soft biomaterials may be useful, as patients with lumbar disc herniation are typically relatively young (<45 years old), and disc cells are expected to remain in these individuals [7,267,268] (Figure 1). However, no suitable biomaterial has been developed to date to replace NP tissue removed after discectomy [168].

**Figure 3:**
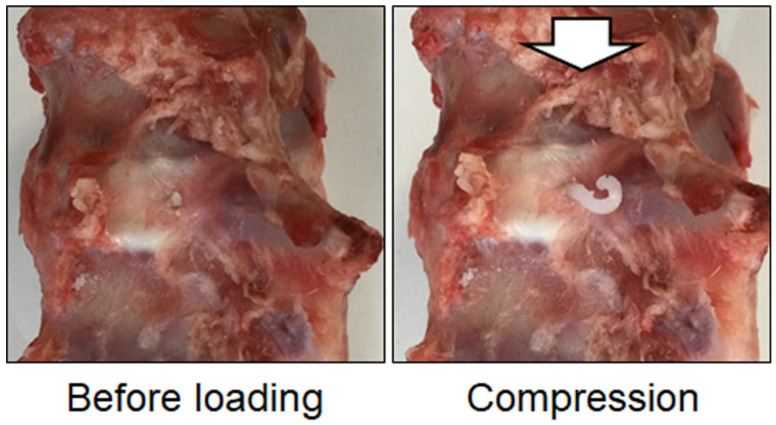
Herniated disc model with a 4.5 mm diameter hole in a pig lumbar disc injected with hydrogel. The hydrogel, which has little adhesive ability, easily extrudes from the disc under compressive load.

Biomaterials for NP regeneration and/or repair after discectomy should not only function to biologically protect cells and promote tissue repair but should also have the following biomechanical functions to address the NP and AF defects. First, the biomaterial should fill the defective area of the NP and AF, have adhesive properties, and not extrude under in vivo loading conditions (Figure 3). Second, the biomaterial should provide structural support in order to restore biological and biomechanical function to the damaged tissues.

### 4.1. Adhesive Function of Soft Biomaterials after Discectomy or AF Injury

Ex vivo and in vivo studies using AF injury models have demonstrated the usefulness of soft biomaterials, such as fibrin, collagen, hyaluronic acid, and alginate-based hydrogels, for IVD regenerative therapy [7,129,140,186,190,192,193,194,205,223,257,260,262,263,269,270,271,272,273,274,275,276]. These hydrogels have the ability to adhere to tissues and allow for in situ repair of disc defects. The adhesion mechanism of hydrogels to tissues can be explained through three modalities, mechanical interlocking, electrostatic interactions, and chemical interactions [257,277,278].

Mechanical interlocking occurs via the biophysical phenomenon in which the roughness of an adherend surface causes the hydrogel to “mate” with the surface irregularities and adhere to the tissue surface [257,278,279]. Meanwhile, electrostatic interaction is a force at the molecular level in which the asymmetrical distribution of attachment surfaces due to differences in the electronegativity of atoms creates partial positive and negative charges between the attachment surfaces, which attract each other making it difficult to functionally separate them [257,279]. Finally, chemical interactions arise from biochemical phenomena acting at the atomic or molecular level and are characterized by diffusion, physisorption, and chemisorption [257,280]. For instance, constituent polymers of the hydrogel network and the biopolymers of the tissue interpenetrate each other at the interface, and diffusion occurs between the polymer adhesive and the adherend [257,281]. In comparison, physisorption is the adhesion of a biomaterial to a tissue substrate by non-covalent intermolecular interactions caused by hydrogen bonding and van der Waals forces at the interface [257,282,283]. Chemisorption is the adhesion of a hydrogel to a tissue substrate by means of multiple types of covalent bonds, such as imine bonds, amide bonds, urea bonds, N-N bonds of hydrazine derivatives, and disulfide bridges, which exist between the adhesive hydrogel and the tissue [257,278,282].

Each type of hydrogel exhibits its own physical and chemical interactions with biopolymers on the tissue surface, and the mechanism of adhesion to IVD tissue differs depending on the hydrogel formulation [257]. The main mechanisms of hydrogel adhesion for IVD repair are proposed to be chemisorption for fibrin-based and collagen-based hydrogels [192,222,257,275], and electrostatic interaction, physisorption, and diffusion for hyaluronic acid-based and alginate-based hydrogels [7,140,205,257].

### 4.2. Evaluation of Biomechanical and Biological Regeneration by Soft Biomaterials in IVD after Discectomy or AF Injury

Candidate biomaterials for IVD repair should be evaluated in ex vivo studies for mechanical feasibility and the associated risk of herniation after in situ application (Figure 3). As noted previously, the risk of herniation after IVD repair can be assessed using cyclic axial loading tests and displacement-controlled ramp-to-failure tests [257]. Fibrin (genipin cross-linked fibrin), collagen hydrogel (riboflavin cross-linked collagen, Rose Bengal cross-linked collagen), alginate (UPAL), chitosan, and cellulose have been shown to improve IVD failure properties and to retain hydrogels after biomechanical loading in ex vivo biomechanical studies, suggesting their clinical usefulness for IVD repair [7,186,192,223,230,233,257,262,269,270,271,272,273] (Table 5).

Histological evaluation in an in vivo AF injury/discectomy model has demonstrated the effect of several soft biomaterials on IVD tissue repair. Meanwhile, treatment with hydrogels, such as fibrin, collagen-based hydrogels (riboflavin-cross-linked collagen and citric acid-1-ethyl-3-(3-dimethylaminopropyl)carbodiimide/N-hydroxysuccinimide collagen gel), hyaluronic acid, and UPAL, has been reported to maintain the IVD structure, retain NP tissue, and cause less degeneration compared with that in disc-injury control groups [7,140,192,222,257,274,275,276,284]. Among the candidate hydrogels, hyaluronic acid-based gels have not been proven to have biomechanical functions in an AF injury model. However, it has been reported for NP augmentation and AF repair that the combination of hyaluronic acid gel with photo-crosslinked collagen gel or fibrin gel prevents disc degeneration in vivo after discectomy [204,223]. These findings indicate a potential need for a combined strategy when it comes to using biomaterials to inhibit disc degeneration after IVD herniation surgery.

### 4.3. Clinical Application of Soft Biomaterial Therapy for IVD Herniation

As previously noted, clinical trials of soft biomaterials for the treatment of disc degeneration are currently limited. In addition, all the clinical trials have been performed using intradiscal injection therapy for patients with no AF defects, and there have been no clinical applications of soft biomaterials for disc repair therapy after discectomy. That noted, a first human pilot study using an acellular bioresorbable UPAL gel in patients with lumbar disc herniation has been conducted [246]. In a preclinical study using a large-animal discectomy model, the UPAL gel not only exhibited sufficient biomechanical properties without protrusion but also histologically promoted disc repair [7]. UPAL gel can be suitable for various shapes of post-discectomy defects while reducing the risk of gel extrusion because the alginate gel can be rapidly cured by covering the AF surface with CaCl2 [7,78,92,168]. Based on current findings, UPAL gel is expected to be clinically applied as a soft biomaterial to safely promote disc repair after disc herniation surgery.

## 5. Conclusions

IVD degenerative disease, which can cause back pain and neurological disorders, is a major obstacle to independent and healthy living by afflicted individuals and is an important issue that needs to be addressed and overcome. However, there is still no effective treatment that can reduce or restore IVD degenerative changes or even alter the course of disease progression. A number of studies have been performed on cell-based IVD regeneration therapies, mainly those using stem cells and biomaterial-based IVD regeneration therapies using tissue engineering techniques. Fortunately, there have been reports of their usefulness as IVD tissue regeneration therapies. In addition, multiple clinical trials evaluating cell therapies, biomaterial therapies, and combinations of these therapies are currently being conducted. In other words, these therapies have the opportunity to become a new and unprecedented treatment for IVD degeneration.

However, many challenges need to be overcome for these regenerative therapies to become clinically practical. With regard to cell-based therapy, there are issues such as the selection of cell types suitable for IVD regeneration therapy, securing quality cells with high proliferative and differentiation potential, problems with cell aging, and securing a sufficient number of cells. Biomaterial-based therapy requires further improvements in biocompatibility and biomechanical functions described in this review, in addition to in vivo stability, biodegradability, and non-immunogenicity. Cost and clinical safety are also important issues for both treatments. Furthermore, the proper indications and patients for these new therapies, as well as the timing of their introduction, need to be fully discussed. Further basic research and preclinical and clinical trials are needed in the future to resolve these issues. With the recent technological innovations in regenerative medicine and tissue engineering, it is expected that IVD regeneration therapy will overcome the current biological, biomechanical, and clinical limitations and ultimately achieve significant improvements in daily activities and quality of life for patients suffering from IVD degenerative diseases.

## Figures and Tables

**Table 1:** Candidate cell sources for intervertebral disc (IVD) regeneration therapy.

Cell Sources		References
Differentiated Cells	IVD-derived cells (nucleus pulposus (NP)-derived cells)	[25,26,27,28,29,30,31,32,33,34,35,36,37,38]
	Chondrocyte-like cells (including chondrocytes derived articular cartilage)	[39,40,41]
Stem Cells	Mesenchymal stem cells (MSCs)	
	Bone marrow-derived MSCs	[26,41,42,43,44,45,46,47,48,49,50,51,52,53,54,55,56,57,58,59]
	Adipose-derived MSCs	[34,60,61,62,63,64,65,66]
	Synovial-derived MSCs	[67]
	Nucleus pulposus-derived MSCs	[68]
	Induced pluripotent stem (iPS) cells	[69,70,71,72,73,74]
	Embryonic stem (ES) cells	[75,76,77]
	Bone marrow aspirate concentrate (BMAC)	[78,79,80]

**Table 2:** Clinical trials on cell-based intervertebral disc (IVD) regeneration therapy.

Cell Type		Mode	Carrier	Administration Method	Indication	n	Outcome	References
Differentiated Cells	Intervertebral disc cells	Autologous	None	Percutaneous injection	Lumbar disc herniation at 12 weeks postoperatively	112	Improvement in pain, disc hydration improved on MRI	[28,36]
	Activated nucleus pulposus cells	Autologous	None	Percutaneous injection	Disc degeneration adjacent to fused disc	9	No progression of disc degeneration	[25]
	Juvenile articular chondrocytes	Allogenic	Fibrin	Percutaneous injection	Degenerative disc disease with low back pain	15	Improvement in pain and clinical indices, and on MRI	[39]
Stem Cells	Bone marrow MSCs	Autologous	Collagen sponge	Percutaneous injection	Lumbar spinal canal stenosis	2	Vacuum phenomenon and motion segment instability improved on radiograph, hydration improved on MRI	[51]
	Bone marrow MSCs	Autologous	None	Percutaneous injection	Chronic low back pain	10	Rapid improvement in pain and disability, hydration improved on MRI	[52]
	Bone marrow MSCs	Autologous	None	Percutaneous injection	Degenerative disc disease with low back pain	5	Self-reported overall improvement, improvement in strength and mobility	[58]
	Bone marrow MSCs	Allogenic	None	Percutaneous injection	Degenerative disc disease with low back pain	24	Improvement in pain and disability, and on quantitative MRI	[59]
	Adipose-derived MSCs	Autologous	Hyaluronic acid	Percutaneous injection	Chronic discogenic low back pain	10	Improvement in pain and clinical indices	[64]
	Bone marrow concentrate	Autologous	None	Percutaneous injection	Discogenic low back pain	26	Improvement in pain and clinical indices	[79,80]

**Table 3:** Candidate biomaterials for intervertebral disc regeneration therapy.

Biomaterials		References
Synthetic Biomaterials	Polylactic acid (PLA), Polyglycolic acid (PGA), Polylactic-co-glycolic acid (PLGA)	[38,63,123,124,125,126,127,128,129,130,131]
	Polyethylene glycol (PEG)	[132,133,134,135,136,137,138,139,140,141,142]
	Polycarbonate urethane (PU)	[143,144,145,146,147,148]
	Poly epsilon-caprolactone (PCL)	[149,150,151,152,153,154,155]
Natural Biomaterials	Alginate	[7,66,78,92,100,128,158,159,160,161,162,163,164,165,166,167,168,169,170,171,172]
	Agarose	[4,173,174,175,176,177,178,179]
	Fibrin	[39,41,49,90,101,180,181,182,183,184,185,186,187,188,189,190,191,192,193,194,195,196]
	Hyaluronic acid	[57,64,132,140,142,173,185,188,197,198,199,200,201,202,203,204,205,206,207,208,209,210]
	Collagen	[65,132,142,197,210,211,212,213,214,215,216,217,218,219,220,221,222,223]
	Chitosan	[66,172,214,218,224,225,226,227,228,229,230,231]
	Carboxymethylcellulose	[232,233]

**Table 4:** Soft biomaterials as candidates for cell-free intervertebral disc (IVD) regeneration therapy.

Composition of Soft Biomaterials	Abbreviation	Clinical Trials/Preclinical	Mechanism of Regeneration	Ref.
Alginate	UPAL (ultra-purified alginate)	Clinical (in progress)/Preclinical (in vivo, rabbit, sheep)	Induction of endogenous NP cells and NP progenitor cells (GD2Tie2 cells), leading to endogenous IVD repair	[7,246]
Collagen	LASCOL (low adhesive scaffold collagen)	Preclinical (in vivo, rat)	Promotion of the formation of cell aggregative spheroids that facilitate the maintenance of the original disc NP phenotype, upregulation of the expression of chondrogenic genes	[220]
Fibrin	Fibrin sealant	Clinical/Preclinical (in vivo, rat)	Suppression of the acute proinflammatory cytokine (TNF-α, IL-1β, IL-6) production, increasing expression of pro-resolution cytokines (IL-4, TGF-β), inhibiting nucleotomy-induced progressive fibrosis of the NP	[192,195]
Hyaluronic acid	HMW HA (high molecular weight hyaluronic acid microgel)	Preclinical (in vivo, rat)	Regulation of inflammation by downregulating IFNα, reduction in cell death by suppressing expression of IGFBP3 and caspase-3 fragment p17, induction of the production of extracellular matrix	[205]

**Table 5:** Biomechanical evaluation of soft biomaterials for intervertebral disc (IVD) repair after a discectomy or annulus fibrosus injury.

Composition of Soft Biomaterials		IVD Model (Ex Vivo)	Biomechanical Evaluation Method	Outcome	References
Fibrin	Genipin cross-linked fibrin	Bovine	Cyclic axial tension–compression, torsion	Full restoration of compressive stiffness, partial restoration of tensile and torsional stiffness	[186]
		Ovine	Cyclic axial tension–compression, torsion	Restoration of axial range of motion and torque range	[269]
		Bovine	Cyclic flexion–extension, torsion, bending	Restoration of torsional stiffness, bending range of motion, low risk of herniation in bending and compression	[270]
		Bovine	Ramp-to-failure test	Low risk of herniation	[271]
Collagen hydrogel	Riboflavin cross-linked collagen	Ovine	Cyclic axial tension–compression, torsion	Restoration of torsional stiffness and torque range (combined with nucleus pulposus augmentation using hyaluronic acid)	[223]
		Rat	Axial compression (uniaxial stress-relaxation)	Improvement in effective equilibrium and instantaneous moduli (combined with nucleus pulposus augmentation using hyaluronic acid)	[273]
		Rat	Axial compression (uniaxial stress-relaxation)	Improvement in effective equilibrium and instantaneous moduli	[272]
	Rose Bengal cross-linked collagen	Rabbit	Cyclic axial compression, torsion push-out test	No extrusion after loading (40,320 cycles with 0.4 to 0.8 MPa compressive loading, 0–25 degree torsion)	[262]
Alginate	Ultra-purified alginate (UPAL)	Ovine	Static axial compression, rotation, flexion–extension, bending, cyclic axial compression	No extrusion after loading (compression loading test up to 1000 N, or 1000 cycles with −300 N to 300 N of axial loading). Partially restored compression stiffness	[7]
Chitosan	Triple-interpenetrating-network hydrogel comprised of dextran, chitosan, and teleostean	Human	Cyclic axial compression	No extrusion after loading (10,000 cycles with 0.12 and 0.96 MPa compressive loading)	[230]
Cellulose	Carboxymethylcellulose	Bovine	Ramp-to-failure test, fatigue endurance test	Reduction in herniation risk compared to injury group, restoration of failure strength, maximum stiffness, and subsidence to failure. Restoration of fatigue endurance compared to injury group.	[233]
